# Remarks upon Inversion of the Uterus*A small portion of this article appeared in the *American Journal*, for July, 1842.—Eds.

**Published:** 1844-01

**Authors:** W. L. Sutton

**Affiliations:** Georgetown, Ky.


### WESTERN JOURNAL

OF

### MEDICINE AND SURGERY.

JANUARY, 1844.

ART. I.—*Remarks upon Inversion of the Uterus.** By W. L. SUTTON, M.D. of Georgetown, Ky.

It is somewhat natural that a physician engaged in extensive practice in any one department of the profession, should conclude that he has seen and is well acquainted with every form and variety of disease of that particular department. Thus we have Mr. Sharpe, and at one time Mr. Benj. Bell, denying that dislocations of the femur ever take place; and Mr. Bell, doubting, to the last, the occurrence of that variety of the dislocation, which the general experience of the profession shows to be most common. Circumstances of this

* A small portion of this article appeared in the *American Journal*, for July, 1842.—EDS.

kind should teach us two useful lessons. First—Not to be too ready to draw sweeping conclusions. Second—That the opinions of those highest in authority, and of the most extensive experience, should still be received with an eye to human fallibility.

I premise these reflections to a few observations, which I propose to offer on Inversion of the Uterus; an accident which Dr. Dewees, the highest obstetric authority in this country, and Drs. Denman, Hunter, and Ford, equally high in England, declare cannot be rectified when complete. That the cases which occurred in Dr. D.'s practice, if taken alone, or rather if *all* cases of inversion were such as occurred in his practice, then I should not doubt the correctness of his precepts. But as I have positive evidence that inversion may occur under more favorable circumstances—circumstances under which the doctor's authority has had a benumbing influence—I am disposed to offer to the profession such observations as my experience and reflections justify.

In the second case detailed in this paper, I returned the flaccid uterus into the vagina and desisted, not because of any insuperable difficulty in completing the reduction, but because I had a distinct recollection of Dr. Dewees' authority, and also of a former case which a long continued attempt had proved unmanageable:—whereas Professor W. H. Richardson, of Transylvania, who arrived perhaps three hours after the accident, readily affected a complete reduction.

I shall in the first place detail two cases which fell under my notice, and then add such remarks as may present themselves. In doing this I shall be compelled to speak much of Dr. Dewees' authority, I trust with that respect, which I feel for him as one of the great lights in our profession, and also with that freedom which the cause of suffering humanity seems to require.

Case I.—November 16, 1823, I was desired to visit Mrs. S., who was said to be in labor. When I entered the room, the midwife observed there was something wrong. Upon making examination, the first thing which attracted my attention was a tumor, which, from its size and firmness, I, for an instant, took to be the head of a child; and supposing the shoulders had engaged the pelvis unfavorably, I ran my finger up the neck to liberate them; when I found that this tumor was a part of the mother, and nothing less than the uterus inverted and expelled. I now first learned that the child had been born. The midwife assured me that she did not have a worse time than common; that the placenta came away in good time, and without difficulty; that the tumor followed it closely; and that not knowing what it was, she did not know whether to permit its exit or not. I endeavored to replace it by grasping it between my hands, and after squeezing it for some time, pushing it in the direction of the outlet of the pelvis. But the tumor was so firm that little impression could be made upon it, and I was utterly unable to reduce it. Upon squeezing the uterus, several blood vessels spouted and bled for a short time. The patient had a ghastly aspect; lips bluish, pupils dilated, pulse very weak: yet the hemorrhage had not been considerable. She did not complain much, but I thought that was owing to the diminution of sensibility. Having become entirely satisfied that longer endeavors to replace the uterus would be fruitless, and must still hasten her dissolution, I desisted, and attempted to sustain her by stimuli, small in quantity and frequently repeated. In this also I failed. Her pulse soon disappeared, and she died in about three hours. This woman was said to be somewhat loose in her morals: had conceived three times and miscarried once; at which time she was said to have had a prolapsus uteri.

Case II.—October 19, 1835, Mrs. H. in labor with her first child. The uterus being expelled with the child, I saw her perhaps in half an hour. The placenta was yet partially attached to the fundus uteri; the body of the uterus completely expelled the vulva; no hemorrhage, great sinking, lips and countenance livid, pulse scarcely perceptible—the uterus not firm as in case 1st. Sent off for Dr. Richardson—separated the placenta, and by moderate and continued pressure returned the uterus into the pelvis. Dr. Richardson, arriving some time afterwards, completed the reduction. She continued very weak and faint, and had frequent retching. This state was considerably altered by injections of starch and laudanum.

In this case, the membranes gave way several hours before the child was born, the presentation natural, pains rather short and at considerable intervals. The body was not expelled by the same pain that expelled the head, but the uterus followed the body by the same pain; the cord was rather short.

Evening.—She has taken small doses of stimuli during the day, also a dose of ol. ricini, which vomited her. Complains of great soreness, pulse weak and very quick, lochia proper; has passed no urine, nor felt any disposition to do so.

20th, 3 o'clock A. M. A great deal of pain in the uterine region; has passed no urine or fæces, nor feels any disposition; pulse small, somewhat hard and very frequent; severe head ache. Drew off about three pints of urine; bled to ℥viii; cold water to head; ol. ricini ℥ii. Evening. Medicine worked well; dejections said to be proper; pulse still frequent (about 150); head ache undiminished; no abdominal pain; lochia have been rather profuse, but at present proper. Sinapisms, which have been applied to the head, having failed to give relief, a blister was applied to the back of the neck; laudanum gtt.x. to restrain the operation of the oil. Discharge of urine natural.

21st.—Head still aches; pulse 132; bowels freely open; urine plenty; lochia proper; no milk. Blister drew well without materially relieving the head.

22d.—Head still aches; skin pleasant; pulse 132; no milk; lochia offensive and pale. ℞ Draw the breasts, and wash the vulva and vagina with chamomile tea.

23d.—Head somewhat relieved; skin pleasant; pulse 132; some appetite; no milk; urine scant; lochia offensive; bowels have not been moved for thirty-six hours; a little tenderness in the uterine region. ℞ Injection of chamomile tea into the uterus and vagina. Seidlitz powders to keep the bowels regular.

25th.—The head has nearly ceased to ache, but feels very sore. The injections into the uterus appear to have benefited her much. No fetor attends the lochia; feels comfortable; skin natural; bowels in good order; no milk, but some soreness of the breasts. From this time she continued to improve, but her health remained delicate for some time. She never had any secretion of milk. In the management of this case I had the benefit of Dr. Richardson's advice, who saw her twice with me after the reduction was affected.

*Causes.*—Dr. Dewees tells us*—"For the uterus to become completely inverted, several circumstances must combine; first, the fundus must most probably contract while the body and neck must be flaccid; second, a force or weight must be applied to the fundus which is capable of making it descend through the os internum: this force may be the power applied to the cord; and the weight may be the placenta itself, when it is engrafted immediately upon the fundus, or the pressure of some of the abdominal viscera."—par. 1269. Again. "The remote cause of this accident is the want of power or disposition in the body and neck of the uterus to contract. This may be occasioned by an over distention of this organ from an excess of liquor amnii; from the unusual size of the fœtus; from a compound pregnancy; from hemorrhagy; from passions or emotions of the mind; from exhaustion in consequence of previous disease; from long continued efforts to effect delivery, &c."—par. 1279. By others, force applied to the cord is considered as the cause, in most instances. I am very far from believing that what I cannot comprehend, must be unintelligible to others. I therefore only express my own opinion when I say that I do not understand the agency or necessity of the first circumstance, in the above mentioned combination, viz: "The fundus must most probably contract while the body and neck must be flaccid." I can very well

* In quoting from Dr. Dewees, I use the fifth edition. In the seventh edition, the latest I have seen, the chapter on inversion is not altered from the fifth.

understand how a contraction of the fibres of the fundus (the body remaining uncontracted), may diminish its convexity; but I do not comprehend how it should cause the fundus to dip into the body. Neither do I see why the contraction of the fundus would render the body less able to support it. We might suppose that the contraction would expel some of the fluids from the walls of the uterus, and thereby remove some of the weight. Again, if the fundus contracts, it thereby detaches the placenta; that is, if we consider its attachment at the fundus, as a necessary requisite to inversion; but in about half the cases which are fully reported, the placenta remained adherent. We are therefore, in my opinion, reduced to the second and only circumstance in Dr. D.'s combination of proximate causes; namely, a force or power applied to fundus uteri capable of making it descend through the os internum. There is no doubt that force applied to the cord sometimes produces inversion. Of this Dr. Meigs furnishes us with a strong example; Dr. Dewees one, in which he very strongly, and most probably, very justly, suspected this as the cause. But Dr. D. disbelieves this as the common cause; and in the two instances which I saw, this was not the cause—at least, so far as the midwife was concerned, and the persons present could be believed—I have myself every confidence in the statements made on each occasion. The midwife who attended the first case, was decidedly not rash, but would request assistance upon any difficulty presenting itself. In the second case, the circumstances and the manner of detailing them, corroborated by declarations of the patient and her friends, left no doubt in my mind as to the correctness of the statement.

I therefore believe that traction applied to the cord is not necessary. But perhaps the voluntary efforts of the patient acting upon a uterus suffering under atony and consequent flaccidity, are frequently the immediate cause of inversion. Although the cord in the second case is mentioned as "rather short," yet it was not unusually so; and my present impression is, that it did not pass round the neck or any limb—yet no mention is made in my notes, by which I can say positively that the enquiry was made.

Of the cause of this atony, we are, and perhaps to a great extent must remain, ignorant. That the circumstances mentioned by Dr. D., to-wit: "over distention of the uterus from an excess of liquor amnii; from an unusual size of the fœtus; from a compound pregnancy; from hæmorrhage; from passions or emotions of the mind; from exhaustion from previous disease; from long continued uterine efforts to effect delivery, &c," may produce atony, I do not question; and I am disposed to believe that the last is the most usual. If, after a violent and protracted uterine exertion, there is, as may reasonably be believed, a corresponding relaxation, the uterus being emptied of its contents, is in a condition very fit to be protruded by the voluntary efforts, continued a little longer than the contractions of the uterus. I am disposed to believe that this is the true solution in my second case. The membranes gave way about three hours before the birth of the child. No mention was made of any unusual quantity of liquor amnii. The child was not above the ordinary size, single. There had been no hemorrhage; no passions or emotions of the mind; no previous disease. And although it was said that there was no inordinate straining, yet as ladies are sometimes encouraged to "help themselves" by voluntary efforts, that may very readily have been overlooked in the last agonies of labor.

This accident has been divided into complete and incomplete. The definition of complete inversion, as given by American authors, is different from that given by European writers; and I conceive more correct. By European writers the inversion is said to be complete when the fundus escapes through the os externum. (Den. par. 1317.) Dewees, on the other hand, defines a complete inversion to consist "in the fundus and body of the uterus passing through the os internum, or its being turned entirely inside out, to the very mouth of this organ." Again, "I have known the inversion to be complete and yet the fundus not escape from the vagina." (Id. 1266.) The incomplete is defined by Dr. D. as one, "in which neither the body nor fundus has escaped through the os uteri." This definition is, I humbly think, much less perspicuous than we should expect from the author—one, indeed, which he forgets or disregards before he finishes the paragraph; where he describes the second degree of partial inversion thus: "where it has passed, perhaps half its length, through the os uteri." And third where "it is completely inverted except a portion of the body and neck." I conceive when half the organ has passed through the os uteri, the fundus "must have entirely escaped through" it. And I think that the difference between his second and third division of incomplete inversion is somewhat indistinct. Again, the incomplete inversion is divided into that in which the protruding part is strangulated by the mouth, and that in which it is free.

*The Symptoms* which should induce us to suspect this accident are "a sudden, severe, and distressing pain in the region of the uterus; and effort to force or bear down; nausea and sometimes vomiting; ghastly aspect; great faintness with more or less hæmorrhage; cold clammy sweats, pulse small, frequent or extinct. A variety of nervous symptoms may also occur of the most distressing kind, arising most probably from the new situation which the abdominal viscera are forced to take, when deprived of the support of the uterus." Although these symptoms should lead us to suspect the presence of inversion, yet examination alone can be depended on for its ascertainment, as the following case will show. In May, 1839, Mrs. C., after a labor of usual duration, was delivered of a son weighing twelve pounds. She remained quite comfortable for some fifteen or twenty minutes, when she was seized with a sense of faintness, difficult and hurried respiration, constant retching, livid, ghastly countenance, cold sweat, weak, quick pulse. Upon examination she was found to have no inordinate hæmorrhage, and the uterus was found well contracted in its proper place. A liberal administration of stimuli, complete rest, and sinapisms to extremities, gradually restored her to a state of comfort, Dr. Ramsbotham also informs us that "collapse occasionally comes on after labor without any apparent cause; the patient becomes extremely ill, the pulse flags, she becomes restless, and only expresses her sufferings by a groan." This he ascribes "to a want of accommodation of the several parts within the belly to each other in the new state of things."

Dr. Dewees thinks that profuse hæmorrhage and severe pain distinguish the incomplete inversion from that which is complete. European writers, also, although not making it a diagnostic mark, speak of the complete inversion not being in all cases attended by profuse hæmorrhage. Let us examine the cases at present available, and see how they support the distinction.

In Dr. Dewees' first case considerable hæmorrhage is mentioned as present; but pain is not. Second case—The expulsion of the placenta was attended by great pain and great flooding; this was on Friday. On Sunday there was a "renewal of flooding and pains resembling labor pains." But the inversion was not determined to be present until Tuesday. Then no mention is made of pain; but there "was a continuance of uterine discharge." This was the case of partial inversion, which, in despair, the Dr. rendered complete; and the woman was almost instantly "relieved from much of the anxiety and faintness she had before experienced." Not a word said about relief from pain. In third case, nothing is said of pain, except that occasioned by pulling at the cord. There is said to have been "some hæmorrhage," and "a considerable flooding ensued" upon removing the placenta. In case fourth, there was "some hæmorrhage, and considerable pain" after the extraction of the placenta. Three hours after, when the Dr. was called, no mention is made of pain being present; but it is perhaps fair to infer that it was. But "the flooding did not appear to be sufficient to account for her present situation." Of case five we have no detail of symptoms except that the patient was almost exhausted by hemorhage and suffering, and almost pulseless. After completing the inversion "she seemed to improve as a moderate reaction ensued; but it was short-lived; she sank and died. These were all cases of partial inversion. In case sixth nothing is said of symptoms, but the "patient was not so much exhausted;" this was a case of complete inversion and did well.

In Dr. Meigs' first case "the hæmorrhage was enormous;"* nothing said of pain; this was a case of complete inversion. Second case is given on hearsay. The hæmorrhage was said to be profuse, and she was thought to be in extreme danger; but nothing further is given as to the symptoms present. The Dr. thinks, apparently from an examination, two years after the accident, that the inversion was complete. In the two cases which I have seen, there was absence of considerable hæmorrhage and pain—both complete. It is right to add that on separating the placenta, in my second case, there was no marked hæmorrhage.

From an impartial review of all these cases, I doubt whether Dr. Dewees' diagnostic will be supported. In the first place, several of his cases of partial inversion are without very considerable hæmorrhage, and where the flooding was worse than usual, a partial adhesion of the placenta existed. Again, in his case of partial inversion rendered complete, he does not say anything of relief from a hæmorrhage, but from anxiety and faintness.

In Dr. Meigs' first case, which was one of complete inversion, it is said expressly that "the hæmorrhage was enormous." The phraseology of this case would, by fair interpretation, invalidate Dr. Dewees' opinion; and yet I think it probable that the facts of the case may be different from what we would be apt to suppose at first sight; because no mention is made of profuse hæmorrhage continuing after Dr. Meigs' arrival; and especially nothing of it upon separating the placenta. I therefore think it probable that the "enormous hæmorrhage" may have taken place whilst the inversion was yet incomplete. So far as two cases would go, those seen by me would show that free hæmorrhage is by no means a necessary attendant on complete inversion. It may be well to compare Dr. Dewees' third case with my second.

---

*Phil. Prac. Mid., art. Inversion.

His was partial—some flooding when he arrived—considerable flooding upon separating the placenta; mine, no considerable hæmorrhage either before or after detaching the placenta. Altogether, I am disposed to think that Dr. Dewees' diagnosis, so far as regards hæmorrhage, will be found entitled to much respect but not decidedly established.

As regards pain as a distinguishing symptom, I do not think his cases establish the claim; as pain is not mentioned as a prominent symptom except in the second and fourth cases. In the second, nothing is said of pain after Sunday morning, two days before Dr. D. saw the patient. His fifth and sixth cases are given in terms too defective to justify any conclusion. In Dr. Meigs' cases nothing is said of pain. In mine, there was no marked pain; but the state of exhaustion, in which both were found, led me to doubt whether freedom from pain was not occasioned by exhaustion of sensibility.

*Treatment.*—Of the treatment of partial inversion I shall say nothing; and in my observations on the treatment of that which is complete, I shall endeavor to show that it has been remedied, rather than offer anything new as to the manner of operating.

I would observe, in the first place, that an inverted uterus may be found in two different conditions: First—Contracted; second—Flaccid.

The condition in which it is found must determine our mode of proceeding. If the uterus be found contracted, then I am ready to agree with Dr. Dewees, that its replacement is utterly impracticable, at least so long as it remains contracted. Whether a large dose of opium would in any case induce a relaxation, I cannot say; Dr. D. is decidedly of opinion that it would not, and that its only effect has been to relieve pain. Yet if I should ever have another such case, I shall unquestionably try it. My hopes of benefit from a large dose of laudanum, would be founded upon the influence which is known to be exerted by it, in rendering the uterus softer, when it is necessary to turn a fœtus in the uterus, after the waters have been some time discharged. It would be a hope, not a confidence. But the condition of the patient would render an opiate proper.

If the uterus is found relaxed, I would in the first place administer a large dose of laudanum—say a teaspoonful; both as a means of removing the state of extreme exhaustion and as a means of preventing the contraction of the uterine fibres; next, if the placenta still adhere (as most probably would be the case), I would separate it; and, thirdly, attempt the reduction. This I would do in one of two ways. First, by grasping the uterus with both hands, the fingers being spread over its surface, pressing it for some time and then directing its body towards the axis of the vagina, as is recommended by Dewees, and practiced by myself. When I had succeeded in lodging the uterus in the vagina, I would press it upwards with the points of two or three fingers, so as to carry the fundus and body through the mouth of the organ. For it is evident that when advanced thus far in the operation, the directions given for the reduction are no longer likely to be efficient. Or, secondly, by pressing the point of one or two fingers against the fundus, and carrying it upwards through the mouth, as was performed by Dr. Meigs. Upon failure by one mode, I would try the other. I would detach the placenta, because its presence would greatly diminish the pliability of the uterus, and considerably impede the reduction; and because it is shown, as far as one case goes, that a placenta may be detached from the inverted uterus, even in a state of atony, without considerable hæmorrhage. I say *one* case, although I presume Dr. Meigs' first case might be cited in addition, as nothing is said about hemorrhage upon removing the placenta. It will be observed, that in his case, the uterus alternately contracted and relaxed, and especially upon handling it contracted, as is observed when in its proper position. In this it differed from both cases which I saw. In my first, it was permanently contracted; in the second, no contractions were observed, even in attempting its reduction. It however contracted soon after its rectification.

A question may arise here: were Dr. Meigs' first case and my second, uteri completely inverted? I must say that I should not suppose any question as to the fact could arise, had not Dr. Dewees labored to prove that an uterus never was restored after being completely inverted, and expressed his belief in the impossibility of its being done. Inasmuch as I consider his definition of complete inversion as the true one, I will try the cases by it. It is contained in par. 1266, *Syst. Mid.*, and runs thus: "By a complete inversion, I mean the passing of the fundus and body through the os internum, or being turned entirely inside out, to the mouth of this organ; when this takes place, the mouth of the uterus is looking upwards, and is within the cavity of the abdomen. But it is not necessary to the complete inversion, that the body and fundus escape through the os externum; as this condition may happen, and yet the uterus be concealed within the vagina." Now, in the cases under consideration, the inversion was either partial or complete. As I consider them perfectly parallel cases, except the hemorrhage and alternate contractions, in Dr. Meig's case, I shall refer principally to the one seen by myself. If I were to say that the fundus extended full six inches beyond the vulva, I am satisfied I should be within the truth; there was no hemorrhage, no pain; no offset, indicating the place where the body escaped through the mouth, was perceived. Take now the Dr.'s definition, which is certainly the correct one, and his symptoms attending complete inversion, and if they do not show the case to have been one of complete inversion, I shall utterly despair of ever proving anything.

My respect for Dr. Dewees prevents my supposing that he would use any unfairness in his criticisms; yet I certainly do not see the correctness and sufficiency of some of his strictures in proving that the cases of Dr. White and Dr. Teallier were not complete inversions. He declares Dr. White's case to have been partial, because the "woman was in great pain and had lost much blood." He adds: "neither of which circumstances attends complete inversion; for it seems to be agreed that there is not much hæmorrhage at this time, and I

know that pain immediatly ceases when it becomes complete, as I shall state presently." "This patient 'was very faint and no pulse could be felt in either arm;' a condition which constantly attends the partial inversion; especially when the mouth of the uterus contracts firmly upon the body, producing a strangulation of the uterus; which was precisely the situation of Dr. W.'s patient, for he declares 'the neck was a little contracted.' Now, it must be obvious, upon a moment's reflection, that if the inversion were complete, the mouth of the uterus cannot be felt; for this part now offers its opening to the cavity of the abdomen and is not tangible by the finger." (Par. 1286—7.)

I am free to admit that I am not aware of any case of complete inversion being characterized by great pain, but Dr. D. has certainly forgotten to redeem his pledge to show a state in which "pain immediately ceases when it (inversion) becomes complete." I understand, when it is said the patient had lost much blood, that we have a very fair right to infer that the bleeding had abated on Dr. W.'s arrival—"she was in great pain and had lost," &c. After the Dr. had completed the inversion in his second case, would it have been fair or true to have said that the "inversion was not complete because she had lost much blood?" Again: "She was very faint and no pulse could be felt in either arm," "a condition which constantly attends a partial inversion, especially if strangulated." Now, I am truly glad that it is not added that great faintness and extinguished pulse do *not* attend complete inversion, or uterus not strangulated. An examination of his own cases would have shown that strangulation of the uterus was not necessary to produce great faintness and exhaustion, or even death itself. And my first case proves that a complete inversion is no infallible indemnity against the same state. Again, where Dr. White says "the neck was a little contracted," Dr. D. considers it conclusive that the inversion was partial and strangulated, because if the inversion were complete, the mouth of the uterus could not be felt. With due submission, I would suppose there would be no difficulty in determining that the neck of the uterus was "a little contracted" by feeling that portion of it which was, at the time, external—even if we could not touch the os tincæ. I have no hesitation in saying that the neck, as well as body of the uterus, was contracted in my first case, and that from actual and careful examination, although I could not insert my finger into the os uteri. But in Dr. Teallier's case, there was, on the 10th day after delivery, "a smooth red tumor of the size of a large pear, the large end resting on the thighs, the small tied in the pelvis. No hæmorrhage and little pain." Why was this declared to be a case of partial inversion? "Because the neck of the uterus is charged with the difficulty of reduction, in the first attempts for that purpose."—Par. 1325. If the neck is not the cause of difficulty in all these cases, in the name of common sense, what is? And lastly, Dr. D. proves that a case, recorded in the New England Journal, is also one of partial inversion. "First—Because there was too much hæmorrhage and too many distressing symptoms. Second—Because the author expressly and conclusively determines this point, by saying the neck of the uterus reached above the pubis."—Par. 1307. To the first branch of the proof, it is not necessary to repeat what was said on the same subject when speaking of Dr. White's case. To the second branch we may oppose one part of Dr. D.'s definition of a complete inversion. "When this takes place, the mouth of the uterus is looking upwards, and is within the cavity of the abdomen." I will not say that these are convertible phrases, but I do think that the difference, if any, is too slight to be laid hold on by a liberal critic. I think that a careful and impartial examination of Dr. D.'s criticisms will result in a conviction, that he has failed to prove that the cases referred to, were not cases of complete inversion. At the same time, I candidly acknowledge that it is only since my second case, and a more thorough consideration of the subject, occasioned by the result of that case, that I feel myself authorized to come to a conclusion different from that of the author.

A few words concerning Dr. Dewees' recommendation to convert a partial strangulated inversion, into a complete one. I expressly disclaim any idea of ability to offer any thing decidedly for or against the proposition; but simply to express some reflections which offer themselves to my mind, upon an examination of the cases above referred to. The object is to relieve the patient from pain, flooding, and the various nervous and distressing symptoms. Its usefulness is founded upon one successful case. But all the cases on record, so far as I know, go to show that all these symptoms attend all cases of inversion, whether partial or complete, strangulated or free; not, to be sure, with equal severity in every case; but Dr. D.'s first case proves that the very worst train of symptoms, and even death itself, may attend a simple dipping of the fundus; and my first shows that the like train of symptoms, and the same result may attend the complete inversion—as I suppose where it was instantaneous, and of course, not strangulated even for a moment. We may suppose it probable that the Dr. himself had no overweening confidence in the remedy, because nearly twenty years after his successful case, he was called to a patient who "was nearly exhausted by hæmorrhage and suffering, and almost pulseless;" yet he "persevered in the attempt at reduction, nearly two hours."—Note to par. 1301. At last he completed the inversion, but the woman died. Thus we see that he truly considered it a dernier resort, and I think we may add "anceps remedium." Perhaps, where there is a strangulated inversion with considerable hemorrhage, upon finding it impracticable to reduce, we might complete the inversion. It is certainly proper to know and recollect what has been the treatment, and result, in as many cases as possible. Burns, in his system of midwifery, refers to the following cases. The appearance of gangrene from strangulation took place. The womb was scarified and the swelling quickly disappeared. The patient got well. In another case, the uterus was mistaken for a polypus, and the ligature applied. The mistake being discovered, it was instantly withdrawn, but the woman died in a few days.

In conclusion, I hope I am distinctly understood as not speaking with authority. I only wish to bring the subject before the profession for their consideration: knowing that they are entitled to the reflections of their most humble member, most especially upon subjects, concerning which the experience of the most exalted must of necessity be very limited.

